# Investigating Genetic Determinants of Plasma Inositol Status in Adult Humans

**DOI:** 10.1093/jn/nxac204

**Published:** 2022-09-02

**Authors:** Eleanor Weston, Faith Pangilinan, Simon Eaton, Michael Orford, Kit-Yi Leung, Andrew J Copp, James L Mills, Anne M Molloy, Lawrence C Brody, Nicholas D E Greene

**Affiliations:** Developmental Biology and Cancer Department, Great Ormond Street Institute of Child Health, University College London, London, United Kingdom; Genetics and Environment Interaction Section, National Human Genome Research Institute, National Institutes of Health, Bethesda, MD, USA; Developmental Biology and Cancer Department, Great Ormond Street Institute of Child Health, University College London, London, United Kingdom; Developmental Biology and Cancer Department, Great Ormond Street Institute of Child Health, University College London, London, United Kingdom; Developmental Biology and Cancer Department, Great Ormond Street Institute of Child Health, University College London, London, United Kingdom; Developmental Biology and Cancer Department, Great Ormond Street Institute of Child Health, University College London, London, United Kingdom; Epidemiology Branch, Division of Population Health Research, Eunice Kennedy Shriver National Institute of Child Health and Human Development, National Institutes of Health, Bethesda, MD, USA; Department of Clinical Medicine, School of Medicine, Trinity College, Dublin, Ireland; Genetics and Environment Interaction Section, National Human Genome Research Institute, National Institutes of Health, Bethesda, MD, USA; Developmental Biology and Cancer Department, Great Ormond Street Institute of Child Health, University College London, London, United Kingdom

**Keywords:** *myo*-inositol, *chiro*-inositol, genome-wide association study, mass spectrometry, glucose, inositol transporter

## Abstract

**Background:**

*Myo*-inositol (MI) is incorporated into numerous biomolecules, including phosphoinositides and inositol phosphates. Disturbance of inositol availability or metabolism is associated with various disorders, including neurological conditions and cancers, whereas supplemental MI has therapeutic potential in conditions such as depression, polycystic ovary syndrome, and congenital anomalies. Inositol status can be influenced by diet, synthesis, transport, utilization, and catabolism.

**Objectives:**

We aimed to investigate potential genetic regulation of circulating MI status and to evaluate correlation of MI concentration with other metabolites.

**Methods:**

GC-MS was used to determine plasma MI concentration of >2000 healthy, young adults (aged 18–28 y) from the Trinity Student Study. Genotyping data were used to test association of plasma MI with single nucleotide polymorphisms (SNPs) in candidate genes, encoding inositol transporters and synthesizing enzymes, and test for genome-wide association. We evaluated potential correlation of plasma MI with d-*chiro*-inositol (DCI), glucose, and other metabolites by Spearman rank correlation.

**Results:**

Mean plasma MI showed a small but significant difference between males and females (28.5 and 26.9 μM, respectively). Candidate gene analysis revealed several nominally significant associations with plasma MI, most notably for *SLC5A11* (solute carrier family 5 member 11), encoding a sodium-coupled inositol transporter, also known as SMIT2 (sodium-dependent *myo*-inositol transporter 2). However, these did not survive correction for multiple testing. Subsequent testing for genome-wide association with plasma MI did not identify associations of genome-wide significance (*P* < 5 × 10^−8^). However, 8 SNPs exceeded the threshold for suggestive significant association with plasma MI concentration (*P* < 1 × 10^−5^), 3 of which were located within or close to genes: *MTDH* (metadherin), *LAPTM4B* (lysosomal protein transmembrane 4 β), and *ZP2* (zona pellucida 2). We found significant positive correlation of plasma MI concentration with concentration of dci and several other biochemicals including glucose, methionine, betaine, sarcosine, and tryptophan.

**Conclusions:**

Our findings suggest potential for modulation of plasma MI in young adults by variation in *SLC5A11*, which is worthy of further investigation.

## Introduction

Nutrient sufficiency is a key determinant of health during development and postnatal life. Among such molecules, numerous functions have been identified for inositol (cyclohexanehexol), a 6-carbon sugar alcohol whose 9 possible stereoisomers form a subgroup of cyclitols of which *myo-*inositol (MI) is the predominant naturally occurring form (1, 2).

MI is incorporated into a wide range of molecules including phosphatidylinositol, inositol phosphates, inositol glycans, and inositol sphingolipids (3, 4). As a result, MI is involved in key physiological functions via several distinct signaling pathways (5–8). For example, classical phosphoinositide signaling involves cleavage of phosphatidylinositol (4,5)-biphosphate (PIP_2_) to generate diacylglycerol and inositol triphosphate (IP_3_), inducers of protein kinase C activity and calcium release, respectively. Further phosphorylation of PIP_2_ generates PIP_3_, which mediates additional signaling, including activation of AKT1 (serine threonine kinase 1). Phosphoinositides play additional roles in regulation of membrane trafficking and protein–membrane interactions. Inositol is also a component of glycosylphosphatidylinositol (GPI) anchors, which mediate protein–membrane attachment or can be cleaved by GPI-specific phospholipases to generate inositol phosphoglycans, which are reported to have second messenger properties, including as insulin mimetics (9).

Dysregulation of inositol availability, inositol phosphate metabolism, or phosphoinositide signaling networks is associated with a range of disorders including neurological and psychiatric conditions, cancers, and Lowe oculo-cerebro-renal syndrome (7, 8, 10). Enzyme components of inositol metabolism therefore represent drug targets in various conditions. Supplemental MI can also have beneficial effects in conditions such as depression, anxiety, and polycystic ovary syndrome, whereas supplementation during pregnancy can prevent or ameliorate gestational diabetes (11–13).

In addition, there is a functional requirement for MI during embryonic development. Inositol deficiency inhibits neural tube closure in mouse embryos, leading to cranial neural tube defects (NTDs), which are also common congenital anomalies of the central nervous system in humans. Conversely, MI supplementation in mice can prevent NTDs in genetic and diabetes-induced models of NTDs (4), and in a mouse genetic model of NTDs induced by folate deficiency (14). Clinical use of MI in pilot studies and a randomized clinical trial suggest that this protective effect can also be replicated in high-risk pregnancies in humans (15, 16). The requirement for MI in neural tube closure is yet to be clearly defined and could potentially involve ≥1 of its intracellular functions. For example, possible involvement of inositol phosphate signaling is suggested by the finding that in mice cranial NTDs can be caused by mutation of *Itpk1* (inositol 1,3,4-triphosphate 5/6 kinase), *Pip5k1c* (phosphatidylinositol-4-phosphate 5-kinase type γ), or *Inpp5e* (inositol polyphosphate-5-phosphatase E), encoding an inositol phosphate kinase, inositol phosphoinositide kinase, and an inositol phosphoinositide phosphatase, respectively (17–19). Moreover, a maternal polymorphism in *ITPK1* might be associated with human NTDs (20).

**FIGURE 1:**
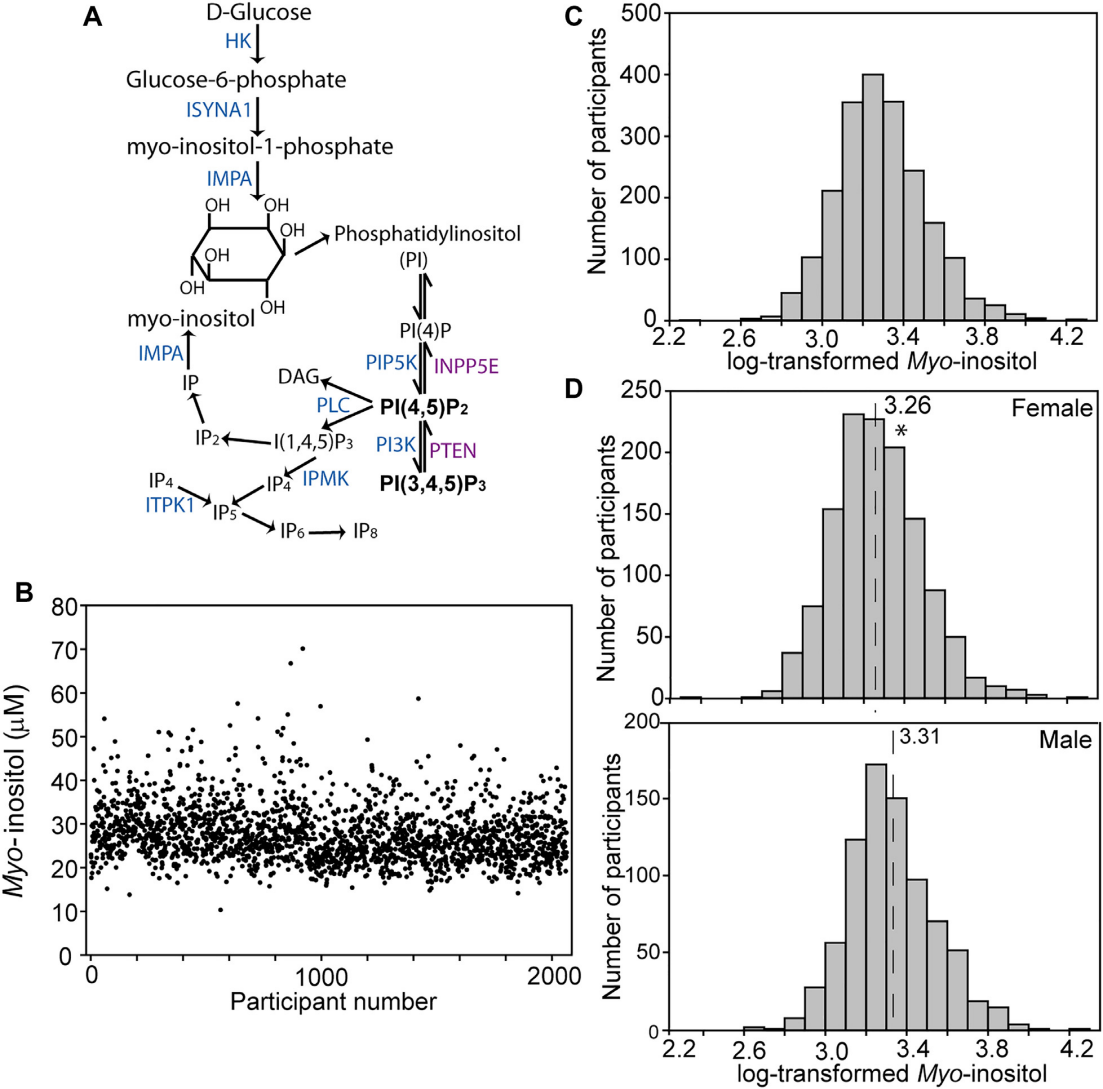
Plasma *myo-*inositol concentration in young adults (Trinity Student Study). (A) Summary of MI synthesis pathway, phosphoinositide signaling, and recycling of inositol phosphates. (B) Concentration of MI in plasma by participant number. (C) Distribution of log-transformed values for MI concentration (all 2064 individuals). (D) Distribution of log-transformed MI values in females and males with median values indicated by dotted line. Mean plasma MI (± SE) was 26.9 ± 0.18 μM in females (*n* = 1258) and 28.5 ± 0.23 μM in males (*n* = 806). *Significantly different from males (*t* test; *P* < 0.0010). DAG, diacylglycerol; HK, hexokinase; IMPA1, inositol monophosphatase 1; INPP5E, inositol polyphosphate-5-phosphatase E; IP, inositol phosphate; ISYNA1, inositol-3-phosphate synthase 1; ITPK, inositol 1,3,4-triphosphate 5/6-kinase; MI, *myo-*inositol; PI, phosphoinositide; PI3K, phosphoinositide 3-kinase; PIP, phosphatidylinositol phosphate; PLC, phospholipase C; PTEN, phosphatase and tensin homolog .

MI status is determined by endogenous synthesis, intake in the diet, uptake and intracellular transport, utilization within tissues, and catabolism (1). De novo synthesis of MI from glucose is achieved in various tissues via sequential phosphorylation to glucose 6-phosphate, conversion to *myo*-inositol 1-phosphate, and dephosphorylation to MI mediated by action of hexokinase, inositol-3-phosphate synthase 1 (ISYNA1), and inositol monophosphatase 1 (IMPA1), respectively (**Figure 1**A) (1, 3, 4). Free MI can also be recycled intracellularly from inositol phosphates by the action of inositol poly- and monophosphatases. It has been estimated that MI synthesis in the human kidney is ∼2 g/d, which exceeds the typical dietary intake of ∼1 g/d (1, 21), and a minimal requirement for dietary MI has not been determined (13). Nevertheless, deficiency or supplementation is sufficient to modulate circulating MI concentrations in various animal models. Despite access to maternal inositol, MI synthesis within the embryo appears essential because *Impa1*-null mice show partially penetrant lethality, which can be rescued by maternal MI supplementation, although surviving mice exhibit behavioral abnormalities (22). In humans, *IMPA1* mutation is associated with intellectual disability and abnormal electroencephalogram (23, 24).

MI catabolism is mediated by *myo-*inositol oxygenase (MIOX) in the kidney, generating glucuronic acid (1, 25). In addition to shared (and potentially competing) use of some transporters, a potential effect of glucose on MI status is further highlighted by the observation that hyperglycemia and/or diabetes are associated with upregulation of *MIOX* (26).

MI and MI derivatives have been the primary focus of research on inositol functions and potential therapeutic application, and d*-chiro-*inositol (DCI) can have similar effects, although effects that are distinct from MI have also been observed. For example, like MI, DCI has been found to be effective in prevention of NTDs in mice (27), and combined treatment of DCI with MI can be beneficial in polycystic ovary syndrome although the ideal ratio and mechanism of action are still to be fully established (28, 29).

Inositol transport is mediated by a proton-coupled inositol transporter [encoded by *SLC2A13* (solute carrier family 2 member 13)] and 2 higher affinity sodium ion–coupled transporters (sodium/*myo*-inositol cotransporter 1 and 2), encoded by *SLC5A3* and *SLC5A11* (30). Both SLC2A13 and SLC5A11 can also transport DCI, and SLC5A11 also transports some other hexoses including glucose. Exposure of the developing embryo or fetus to inositol is dependent on maternal circulating inositol and transport across the yolk sac and placenta (30). At preimplantation stages, MI uptake is predominantly by sodium-coupled transport, and subsequently all 3 inositol transporters are expressed in the human yolk sac and placenta (30). Hence, all 3 transporters can contribute to support of MI-dependent processes required for embryonic development. In mice, loss of *Slc5a3* results in MI depletion in the fetus and early postnatal lethality (31, 32).

Other than intake in the diet, the transport, synthesis, and metabolism of MI represent multiple possible points at which inositol status could be modulated by functional variation in the molecular mediators of these processes. The combination of biochemical assays with genome-wide association studies (GWASs) offers potential to identify genetic determinants of nutrient status (33–35). In the current study, we adapted MS-based methodology to enable high-throughput quantification of inositol in plasma and examined potential genetic associations with plasma MI. Owing to the relation of MI with glucose, and therefore other metabolites related to central carbon and 1-carbon metabolism, we also evaluated the potential correlation of MI with glucose and other metabolites, as well as DCI.

## Methods

### Study participants

Samples were collected as part of the Trinity Student Study (TSS), in which a cohort of healthy young adults (aged 18–28 y) were recruited at Trinity College Dublin over a 1-y period (2003–2004), as described previously (36–38). Briefly, 2508 individuals were eligible to participate, gave written informed consent, and completed a questionnaire including parameters of age, sex, height, weight, dietary habits (meat eating, vegetarian, or vegan), micronutrient intake from supplements and fortified foods, usual alcohol intake, smoking status, and contraceptive use among female participants. A nonfasting blood sample was collected for metabolite quantification and extraction of DNA. Blood samples were collected into serum tubes and EDTA-coated tubes for measurement of metabolites, processed, and frozen at −80°C within 3 h of collection, and stored until analyzed. Ethical approval was obtained from the Dublin Federated Hospitals Research Ethics Committee, affiliated with Trinity College Dublin. The study was reviewed by the Office of Human Subjects Research at the NIH and by the Research and Innovation Office at UCL Great Ormond Street Institute of Child Health.

### Quantification of MI and DCI

Among TSS samples, sufficient plasma was available for analysis of inositol in 2064 individuals. MI and DCI were analyzed by GC-MS, by adaptation of a published method, with generation of a hexa-acetyl derivative of inositol (39). In addition to inositol enantiomers, this method also allowed simultaneous quantification of glucose in plasma samples.

### Sample preparation and derivatization

Plasma samples (50 μL) were mixed with 50 μL internal standard (100 μM *myo*-inositol-d_6_, 12.5 mM glucose-d_2_; Sigma) and 1 mL methanol, and centrifuged for 5 minutes, 10,000 g at room temperature. Supernatants were collected, dried by lyophilization, and stored at −70°C prior to analysis. Samples were derivatized by addition of 100 μL of 1 mg/ml 4-(dimethylamino)pyridine (Fluka) and 100 μL acetic anhydride, and incubation at 80°C for 30 min. A series of internal standards (0–200 μM MI and 0–25 mM glucose) were prepared and derivatized as above after addition of internal standard solution.

### GC-MS

Samples were analyzed on a GC-MS system comprising Triplus sample autosampler, Trace GC Ultra Gas Chromatography, DSQII mass spectrometer, operated with XCalibur V 3.0.63 software (all ThermoFisher Scientific). GC used an Rxi-5Sil MS fused silica (5% diphenyl/95% dimethylpolysiloxane), 30 m × 0.25 mm internal diameter, 0.25-μm film thickness column (RESTEK). The inlet temperature, MS transfer line, and ion source were set to temperatures of 280°C, 250°C, and 225°C respectively. The split ratio was 1:12. The carrier gas (helium) was set to a flow rate of 0.8 mL/min. Detection was in positive chemical ionization mode with reagent gas (methane) at 2 mL/min. GC-MS was performed using a temperature gradient from 200 to 320°C. Inositol enantiomers (*myo*, d-*chiro, allo, muco*, and *scyllo*) were separated on the basis of retention time (**Supplemental Figure 1**). The glucose in the sample was derivatized and showed different retention time to inositol (**Supplemental Figure 2**).

### Analysis

Selected ion monitoring was used for collecting data on inositol enantiomers (*m*/*z* 372.5–373.5), *myo*-inositol-d_6_ (378.5–379.5), glucose (330–331.5), and glucose-d_2_ (332.5–333.5). Peak areas corresponding to MI, DCI, MI-d_6_, glucose, and glucose-d_2_ were determined by peak integration. Standard curves were constructed by plotting the ratio of inositol (MI or DCI)/MI-d_6_ and glucose/glucose-d_2_ (**Supplemental Figure 3**).

Samples were analyzed in batches of 100–200. As an internal quality control, 2 pools of plasma were prepared with inositol concentrations toward the upper and lower ends of the range. These samples were aliquoted and frozen for replicate analysis in each GC-MS run, for determination of between-day (interassay) variance (CV was ≤7.8% for MI).

### Genotyping

To ask whether there is a genetic influence on concentration of MI in plasma, we made use of the curated SNP genotyping dataset from the TSS cohort (33). Genotyping, quality control, and preparation of the data have been described in detail elsewhere (33). Briefly, DNA samples from participants with high-quality DNA (*n* = 2490) were genotyped using Illumina 1 M HumanOmni1Quad_v1-0_B chips at the Center for Inherited Disease Research (Baltimore, MD). The call rate was ≥98% and minor allele frequency >0.01. The final, clean dataset consisted of genotype data for 757,577 single nucleotide polymorphisms (SNPs) for 2232 participants. This has previously allowed study of genetic associations with multiple metabolites in plasma and serum in the TSS cohort (33).

### Biochemical analyses

Methionine (interassay CV = 3.4%), serine (interassay CV = 5.7%), glycine (interassay CV = 3.3%), and cystathionine (within-day CV = 4–6%) in serum were measured by GC-MS, serum sarcosine and tryptophan (interassay CV ≤4.8%) were measured by GC-tandem MS, and serum betaine (within-day CV = 4–6%) and dimethylglycine (interassay CV <10%) were measured by LC tandem MS. These measurements were performed by Bevital (www.bevital.no) (40). The remaining metabolites were measured as previously described. Briefly, serum folate, red cell folate, and serum vitamin B-12 were measured by microbiological assay (41, 42), with between-assay CVs <11.0%. Serum holotranscobalamin was measured using an AxSYM analyzer (43); the between-assay CV was <11.1%. Serum methylmalonic acid and homocysteine were measured by automated isotope-dilution GC-MS as previously described (33), with interassay CVs of 8.1% and 2.2%, respectively. Formate (interassay variance of 7.4%) was measured in serum by GC-MS, as previously described (35).

### Statistical analysis

Statistical analysis was performed using OriginPro 2019 and SigmaStat v3.5 software. Mean plasma concentrations were compared between sexes by *t* test. Median plasma concentrations were compared between smoking/nonsmoking and oral contraceptive/nonuse groups by Mann–Whitney rank sum test. The log-transformed concentration of MI showed a normal distribution. Because other metabolites did not all show a normal distribution, Spearman rank correlation was used to test for correlation between MI and other metabolites.

Log-transformed MI measurements were used to identify genome-wide association signals. Linear regression with an additive genetic model incorporating age and sex as covariates was performed in PLINK v1.07 (http://zzz.bwh.harvard.edu/plink/) (44). Genome-wide associations were considered significant at *P* < 5 × 10^−8^ or suggestive at *P* < 1 × 10^−5^. Ten candidate genes were selected based on potential involvement in inositol transport, synthesis, and catabolism. Variants within each candidate gene and its 10-kb flanks were considered. Measures of linkage disequilibrium (*D*′, *r*^2^) were generated and tagSNPs (*r*^2^ < 0.8) were identified based on genotyping data from the TSS using Haploview (https://www.broadinstitute.org/haploview/haploview) (45). This approach allows the evaluation of multiple SNPs in or near the same gene, while being able to discern the presence of independently acting SNPs. Significance was set at α = 0.05 for all statistical tests. Evaluating significance in the context of multiple tests was performed by defining tagSNPs (*r*^2^ < 0.8) as independent, and using Bonferroni correction to adjust the threshold for significance in each of the candidate gene groups.

## Results

### Inositol quantification

Sufficient samples were available for determination of the concentration of MI by GC-MS in plasma from 2064 participants in the TSS (Figure 1B). Plasma MI concentration ranged from 10.3 to 83.4 μM (mean 27.6 ± 6.4 μM; median 26.6 μM) and log-transformed values showed a normal distribution (Figure 1C). This range of values was consistent with typically reported concentrations and plasma MI values (26–43 μM), which we previously determined using LC-MS/MS methodology in a control group of healthy adults (30, 46).

Among the study cohort of 1258 females and 806 males, we observed a significant difference in mean plasma MI between sexes, with a shift toward higher values in males (28.5 ± 0.2 compared with 26.9 ± 0.2 μM; *P* < 0.001) (Figure 1D). Correlating with this observation, we found positive correlations of plasma MI with height and weight, but not with BMI, in the overall cohort (but not within sexes) (**Supplemental Table 1**). There was also a correlation of plasma MI with age in the overall cohort (**Table 1**). On the basis of these findings, sex and age were included as covariates in genetic association studies. No association with plasma MI concentration was found for alcohol intake, smoking, or use of oral contraceptives (in females) (Tables 1 and **2**).

**TABLE 1:** Correlation of plasma *myo*-inositol with BMI, age, and alcohol intake in young adults (Trinity Student Study)^1^

Parameter	Mean ± SD	*Myo-*inositol (Spearman ρ)	*P*
BMI, kg/m^2^	23.0 ± 3.1	0.012	0.600
Age, y	22.4 ± 1.7	0.072	0.001^2^
Alcohol intake, g/d	24.4 ± 21.1	0.058	0.0082

1 Correlation was determined using raw MI data in the cohort of 2064 individuals (1258 females and 806 males). MI, *myo*-inositol.

2 Indicates significant correlation (*P* < 0.0023 set as level of statistical significance accounting for Bonferroni correction).

**TABLE 2:** Plasma *myo-*inositol (MI) concentration did not differ with smoking or use of contraceptives in young adults (Trinity Student Study)

Group	Participants, *n* (%)	Median plasma MI,^1^ μM
Total cohort	2059	26.6 ± 0.18
Smoking	648 (31.5%)	26.5 ± 0.30
Nonsmoking	1411 (68.5%)	26.6 ± 0.22
*P*		0.448^2^
Total women	1258	25.9 ± 0.22
Oral contraceptive use	339 (26.9%)	25.7 ± 0.43
No oral contraceptive	919 (73.1%)	26.1 ± 0.26
*P*		0.434^2^

1 Totals represent participants for whom information was provided and median values of MI concentration are shown (± SE of the median).

2 No significant difference between smoking and nonsmoking groups or between contraceptive use and nonuse groups (Mann–Whitney test).

### Investigating genetic association with plasma MI status

SNP genotype data were available for 2064 individuals for whom MI was determined. We first investigated candidate genes selected on the basis of encoding proteins involved in inositol transport, as well as glucose transporters owing to the potential antagonistic interplay between inositol and glucose transport (**Table 3**). Genotyped SNPs within 10 kb of these genes were extracted for candidate gene analysis, encompassing 176 SNPs, of which 106 were considered independent tagSNPs (*r*^2^ < 0.8).

**TABLE 3:** Candidate genes tested for association with plasma MI concentration in young adults (Trinity Student Study)^1^

Gene symbol	Protein	Function	SNPs, *n*	tagSNPs, *n*
*Candidate genes tested for association with plasma MI*				
*SLC2A13*	Solute carrier family 2 member 13	Inositol transport	113	65
*SLC5A11*	Solute carrier family 5 member 11	Inositol transport	31	24
*SLC5A1*	Solute carrier family 5 member 13	Glucose transport	18	10
*SLC5A2*	Solute carrier family 5 member 13	Glucose transport	5	3
*SLC5A3*	Solute carrier family 5 member 13	Glucose/inositol transport	9	4
*Candidate genes relating to inositol synthesis and catabolism*				
*ISYNA1*	Inositol-3-phosphate synthase 1	MI synthesis	7	3
*IMPA1*	Inositol monophosphatase 1	MI synthesis and IP recycling	17	8
*MIOX*	*Myo*-inositol oxygenase	MI catabolism	13	13
*Candidate genes relating to production of highly phosphorylated inositol*				
*ITPK1*	Inositol-tetrakisphosphate 1-kinase	Inositol phosphate kinase	48	36
*IMPK*	Inositol polyphosphate multikinase	Inositol phosphate kinase	17	8

1 Data for individual SNPs in candidate genes are provided in **Supplemental Table 2**. IP, inositol phosphate; MI, *myo*-inositol; SNP, single nucleotide polymorphism.

Among these candidate genes, the most striking finding was for *SLC5A11*, for which nominal association with plasma MI was found for 4 SNPs (RS234605, *P* = 0.0247; RS1718990, *P* = 0.0032; RS28540434, *P* = 0.0008; RS4788439, *P* = 0.0015); the latter 3 SNPs are not independent (*r*^2^ > 0.8). However, none of these SNPs showed significant association with MI, following adjustment of the threshold for significance to account for multiple tests (106 independent SNPs; Bonferroni-adjusted *P* value <0.0005 by study), although RS28540434 approached significance. Nominally significant association with MI (*P* < 0.05) was also found for 5 SNPs in *SLC2A13*, and 1 SNP in each of *SLC5A1* and *SLC5A2* but none survived correction for multiple testing.

We also assessed additional candidate genes related to inositol synthesis (*ISYNA1, IMPA1*) and catabolism (*MIOX*). Among 37 SNPs in these genes, 24 SNPs represented independent tagSNPs (*r*^2^ < 0.8). A nominally significant association between MI and *ISYNA1* (*P* < 0.05 for each of 6 SNPs; represented by 2 tagSNPs) was identified but was not significant following correction for multiple testing. We also selected 2 genes encoding enzymes that are responsible for production of highly phosphorylated inositol (Table 3). These include *ITPK1*, encoding an inositol triphosphate kinase, which has a possible association with NTDs, and *IMPK* (inositol polyphosphate multikinase). Among 65 SNPs in these 2 genes, 44 SNPs represented independent tagSNPs (*r*^2^ < 0.8). Nominally significant association with MI (*P* < 0.05) was found for 2 SNPs in strong linkage disequilibrium (*r*^2^ = 0.93) in *ITPK1* but did not survive correction for multiple testing. Hence, we did not find strong evidence to suggest that common variation in these genes influences circulating MI concentrations.

**FIGURE 2:**
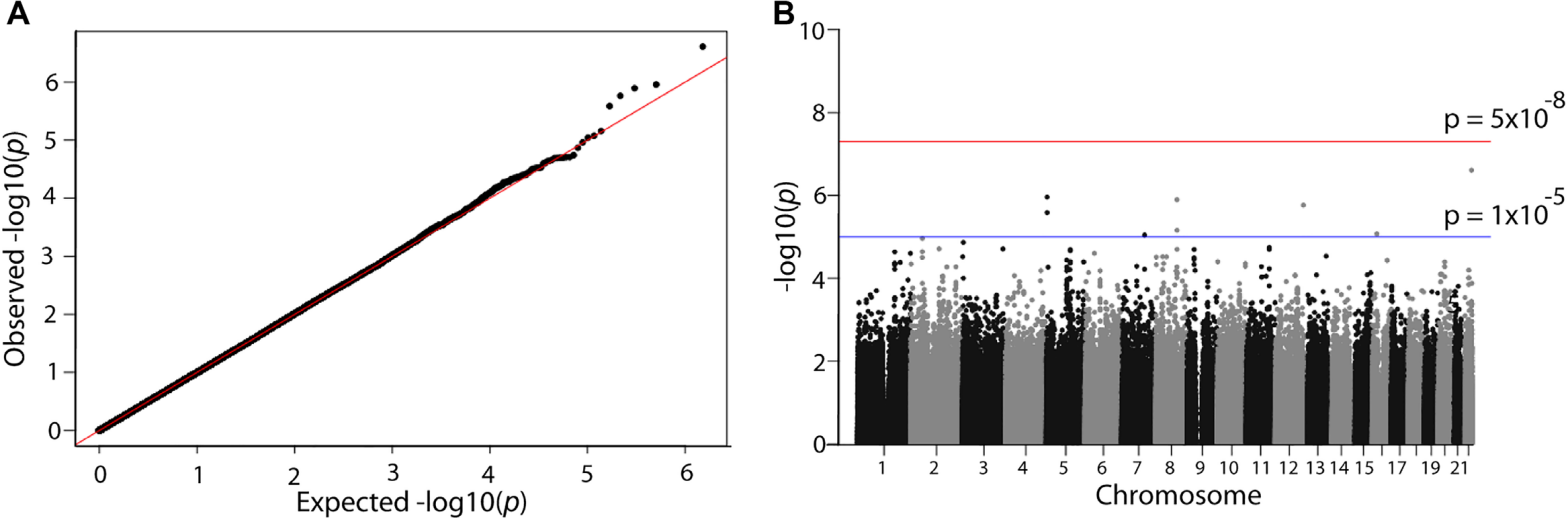
Genome-wide association study of plasma *myo-*inositol in young adults (Trinity Student Study). (A) Quantile-quantile (Q-Q) plot shows distribution of observed vs. expected *P* values. (B) Manhattan plot showing −log_10_(*P*) for each single nucleotide polymorphism ordered by chromosome, indicated by alternating black and gray shading. Lines indicate cut-off for genome-wide statistical significance [*P* = 5 × 10^−8^; line at –log_10_(*P*) = 7.3] and suggestive significance [*P* = 1 × 10^−5^; line at –log_10_(*P*) = 5.0]. Data were obtained from 2064 participants.

We next tested for genome-wide association with MI concentration. A quantile-quantile (Q-Q) plot of *P* values for SNP associations with log-transformed MI, adjusted for age and sex, did not show deviation between expected and observed values, until the very lowest observed *P* values, suggesting that there are no major confounders in the data (**Figure 2**B). Statistical significance in the GWAS is shown in a Manhattan plot (Figure 2A). No SNPs showed *P* values above the threshold for genome-wide statistical significance (*P* < 5 × 10^−8^). However, 8 SNPs exceeded the threshold that defined suggestive significant association with MI concentration in plasma (at *P* < 1 × 10^−5^) (**Table 4**). The genes associated with 3 of these SNPs encode metadherin (*MTDH*, chromosome 8), lysosomal protein transmembrane 4 β (*LAPTM4B*, chromosome 8), and zona pellucida 2 (*ZP2*, chromosome 16). Additional SNPs that showed suggestive significance were in intergenic regions (>10 kb from the closest annotated genes).

**TABLE 4:** SNPs showing suggestive significant association with plasma *myo*-inositol in GWAS in young adults (Trinity Student Study)^1^

Chromosome	SNP	bp	Allele^2^	β^3^	*P*	Gene^4^
5	RS13436726	3,308,137	G	0.0748	1.10 × 10^−6^	—
5	RS10037610	3,321,365	A	0.0704	2.59 × 10^−6^	—
7	RS342301	106,164,564	T	0.0140	9.08 × 10^−6^	—
8	RS2448193	98,716,623	A	0.0311	6.96 × 10^−6^	*MTDH*
8	RS2002450	98,856,971	T	0.0290	1.28 × 10^−6^	*LAPTM4B*
12	RS28390364	131,460,020	A	0.0592	1.73 × 10^−6^	—
16	RS7189430	21,117,507	C	0.0130	8.44 × 10^−6^	*ZP2* ^4^
22	RS135374	45,961,150	T	0.0204	2.46 × 10^−7^	—

1 GWAS, genome-wide association studies; *LAPTM4B*, lysosomal protein transmembrane 4 β; *MTDH*, metadherin; SNP, single nucleotide polymorphism; *ZP2*, zona pellucida 2.

2 Allele is minor allele.

3 β = regression coefficient.

4 Among the 8 SNPs which show suggestive significance (*P* < 1 × 10^−5^), RS2448193 lies at −8.95 kb from *MTDH*, RS2002450 lies at −0.013 kb from *LAPTM4B*, and RS7189430 lies within the *ZP2* gene.

### Correlation of MI concentration with DCI and other metabolites in plasma

**FIGURE 3:**
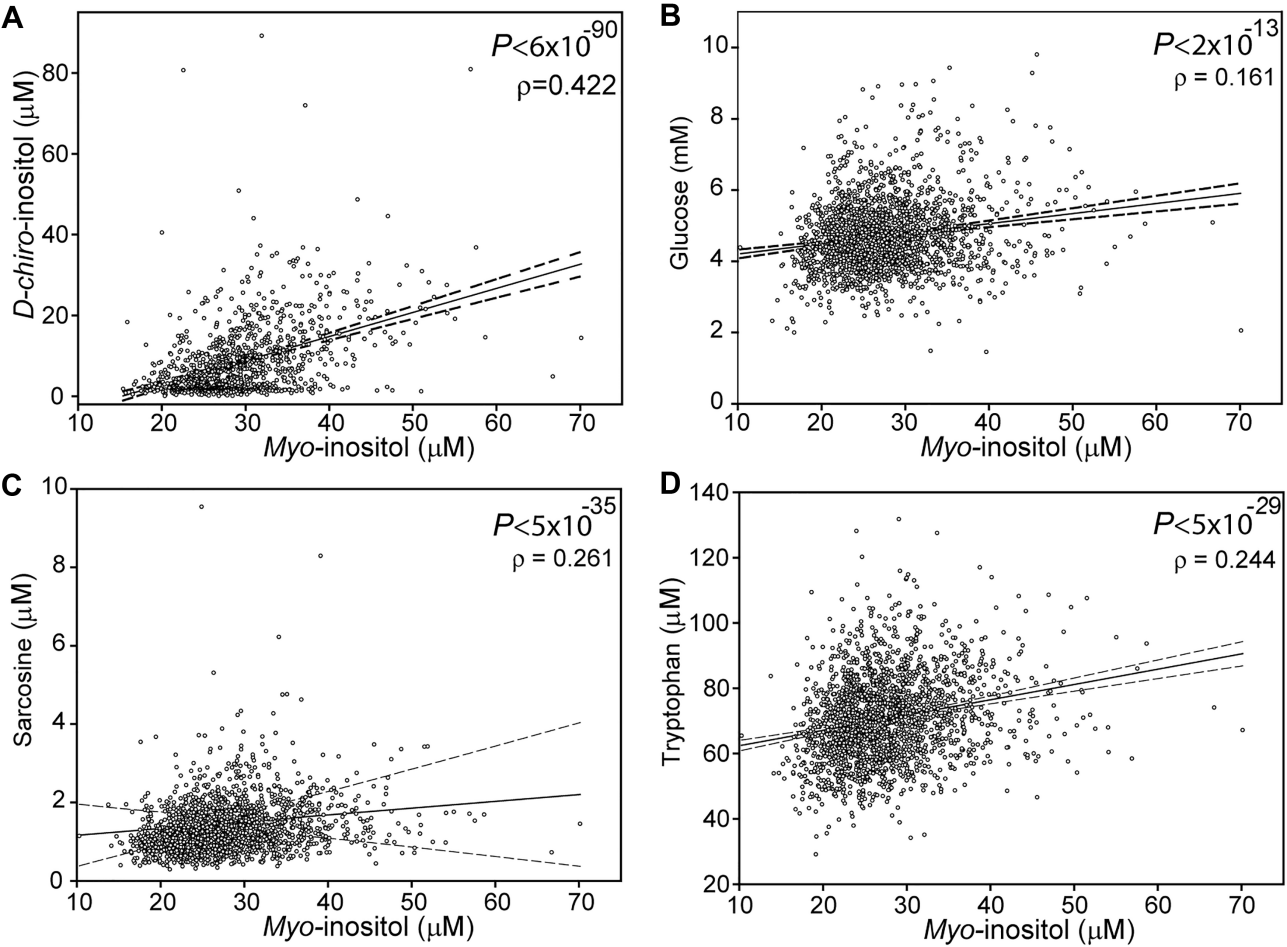
Correlation of plasma *myo-*inositol with d-*chiro* inositol, glucose, sarcosine, and tryptophan in young adults (Trinity Student Study). Plasma MI concentration shows significant correlation with (A) DCI, (B) glucose, (C) sarcosine, and (D) tryptophan. Solid line represents regression line and dashed line indicates 95% CIs. The number of participants was 1066 for MI vs. DCI and 2064 for MI vs. other metabolites. *P* values for Spearman rank correlation and Spearman ρ are shown in each panel. DCI, d-*chiro*-inositol; MI, *myo-*inositol.

Among individuals for whom MI was determined, DCI concentration in plasma was above the limit of detection for 1066 of the samples. The mean plasma concentration of DCI among these samples was 8.5 ± 9.2 μM (with a range from 0.04 to 89 μM, median 5.6 μM) compared with 29.5 ± 6.8 μM (range 15.4 to 70.3 μM, median 28.4 μM) for MI in this group. GWAS analysis did not reveal potential genetic modifiers of DCI with genome-wide or nominal significance. However, DCI concentration showed a significant positive correlation with MI (*P* < 6 × 10^−90^, Spearman rank correlation) (**Figure 3**A).

Owing to the biochemical link between MI and glucose as well as sharing of some transporters, we next asked whether MI status correlates with nonfasting glucose (Figure 3B). We also examined possible correlation with other metabolites. We particularly focused on metabolites linked to 1-carbon metabolism owing to the potential biochemical relation between MI and serine, which share a biosynthetic precursor in glucose 6-phosphate (**Table 5** and examples in Figure 3C, D). Interestingly, we observed correlation of MI with glucose, methionine, cystathionine, betaine, dimethylglycine, sarcosine, tryptophan, serum and red cell folate, glycine, formate, and holotranscobalamin but not serum vitamin B-12, serine, methylmalonic acid, or homocysteine.

**TABLE 5:** Correlation of circulating MI with DCI and other metabolites in young adults (Trinity Student Study)^1^

Metabolite^2^	Concentration (mean ± SD)	Spearman ρ	*P*
*Chiro*-inositol, μM	8.5 ± 9.2	0.422	5.8 × 10^−90*^
Glucose, mM	4.7 ± 1.0	0.161	1.8 × 10^−13*^
Serum folate, nM	22.4 ± 1.7	0.128	4.7 × 10^−9*^
Red cell folate, nM	1070 ± 427	0.117	1.1 × 10^−7*^
Serum vitamin B-12, pM	332 ± 147.0	0.030	0.169
Serine, μM	147 ± 24.0	0.048	0.029
Glycine, μM	293 ± 64.1	0.080	2.9 × 10^−4*^
Formate, μM	30.0 ± 13.6	0.203	1.1 × 10^−20*^
Holotranscobalamin, pM	59.3 ± 31.2	0.090	4.7 × 10^−5*^
Methylmalonic acid, μM	0.2 ± 0.1	0.064	0.004
Homocysteine, μM	8.6 ± 3.0	0.054	0.014
Methionine, μM	29.3 ± 8.3	0.206	4.2 × 10^−21*^
Cystathionine, μM	0.14 ± 0.08	0.203	2.3 × 10^−20*^
Betaine, μM	37.5 ± 14.2	0.230	4.3 × 10^−26*^
Dimethylglycine, μM	4.2 ± 1.2	0.108	8.0 × 10^−7*^
Sarcosine, μM	1.5 ± 6.3	0.261	3.3 × 10^−33*^
Tryptophan, μM	70.6 ± 13.1	0.244	3.7 × 10^−29*^

1 Potential correlation of MI with other metabolites was examined by Spearman rank correlation using raw data, with statistical significance (*) set at *P* < 0.0023 following Bonferroni correction for multiple testing. The number of individuals was 1066 for MI vs. DCI and 2064 for MI vs. other metabolites. DCI; d-*chiro*-inositol; MI, *myo-*inositol.

2 Analytes were analyzed in plasma unless otherwise noted (e.g., serum folate and vitamin B-12 and RBC folate).

## Discussion

*Myo-*inositol, via phosphoinositides and inositol phosphates, plays a key role in numerous cellular functions and is implicated in a range of diseases. We found a small but significant difference in plasma MI in females and males. The biological significance of this difference is as yet unknown. High-throughput analysis of plasma MI by GC-MS provided an opportunity to investigate potential genetic modulators of MI status. Key candidate genes included those encoding the sodium-coupled (SMIT1 and SMIT2) and proton-coupled (HMIT) active MI cotransporters, encoded by *SLC5A3, SLC5A11*, and *SLC2A13*, respectively (47). Among these candidate genes, 4 SNPs covered by 2 tagSNPs and linked to *SLC5A11* were nominally associated with plasma MI (e.g., *P* = 0.0008 for rs28540434). *SLC5A11* is expressed in the gastrointestinal tract and kidney and has been found to be responsible for apical MI transport in the rat intestine (48, 49). Hence, variation in expression or function of this gene represents a plausible mechanism by which plasma MI could be modu-lated.

Another *SLC5A11* variant, rs11074656, has been examined for potential effects on blood MI in children with spina bifida and their mothers (50). Although MI did not differ by analysis of all genotypes, a subanalysis suggested an association of the TT genotype (encoding V182A) with lower MI compared with the CC genotype among the mothers (50). No genetic association of this polymorphism with spina bifida has been reported to date but evaluation of *SLC5A11* in larger-scale studies, that also include cranial NTDs, could be warranted.

Unbiased investigation of potential genetic modifiers of plasma MI by GWAS did not reveal loci of genome-wide significance. However, we found nominally significant associations of plasma MI with SNPs located in or near 3 genes. *MTDH*, which encodes metadherin, also known as *AEG1* (astrocyte elevated gene 1) or *LYRIC* (lysine-rich CEACAM-1 co-isolated protein), is a transmembrane protein that has been implicated in multiple biological processes (51, 52). In particular, MTDH is overexpressed in many cancers, including liver, brain, and breast cancers, and is associated with metastasis and tumor progression with a direct role being indicated by experimental models in mouse (51, 53). In addition to cancer, overexpression of MTDH has been noted in CNS disease including Huntington disease and migraine (52, 54). MTDH can act via multiple downstream pathways with functional effects having been identified in Wnt/β-catenin, NF-κB, MAPK/ERK, retinoid, and PI3-kinase/Akt signaling (53). Although functionally linked to PI3-kinase signaling it is unclear whether MTDH could affect MI status, but the pleiotropic effects of MTDH suggest multiple possible mechanisms by which such an effect could occur. It is not yet clear whether and how plasma MI is associated with cancer risk, but supplemental MI and inositol hexaphosphate (phytic acid; IP_6_) have been suggested to be protective against several cancer types, including colorectal, colon, mammary, and liver (2).

LAPTM4B is a transmembrane protein involved in endosomal sorting and regulation of proteins such as EGFR (epidermal growth factor receptor), whose degradation it suppresses (55). LAPTM4B overexpression has been identified as a prognostic marker in various cancers, with potential effects via modulation of cell proliferation and/or autophagy (56–58). A potential interaction between LAPTM4B and phosphoinositide metabolism is shown by the finding that it can bind and is regulated by the endosomal PIP kinase, PIPKIγi5, and its product PI(4,5)P_2_ (55), providing a means which by EGFR trafficking is modulated.

The other gene with nominally significant association with plasma MI, *ZP2*, encodes one of the glycoprotein components of the zona pellucida, the extracellular matrix that surrounds the mammalian oocyte. Human ZP2 appears to have a particular function in sperm-oocyte binding (59, 60), and mutation can cause infertility. Although MI signaling, via the IP_3_ receptor, is involved in oocyte maturation, and inositol status is thought to modulate fertility in females and males (12, 61), it is not clear how a *ZP2-*associated variant could affect circulating MI.

Overall, our study did not indicate a strong genetic modifier of plasma MI. We speculate that tissue MI could be subject to greater influence of genetic variation as concentration in tissues can be significantly higher than blood owing to active transport (47). For example, some tissues are reported to have a higher intracellular MI concentration than plasma, including brain, pituitary, and kidney, suggesting tissue-specific regulation (21, 25). The relative contributions of endogenous synthesis (e.g., active in kidney) and MI from dietary sources also vary between tissues. Although dietary intake can account for less than half of MI tissue content, this source is likely to vary widely between individuals. Dietary MI comes from free MI, which is abundant in citrus fruits, from IP_6_ (which is enriched in nuts and seeds), and from inositol-containing phospholipids present in animal and plant tissue. We did not have dietary data on the TSS cohort that would have allowed us to consider this variability.

In addition to potential genetic modulators, we also examined potential correlation of plasma MI with other metabolites and nutrients. Correlation, rather than regression analysis, was performed to encompass the possibility that plasma MI influenced or was influenced by the other variable or that both were influenced by a third variable. Each of the correlations that we observed were positive. Although *r* values were generally low, this was not unexpected, given the inherent diversity in a human cohort and use of nonfasting samples. Nevertheless, the sample size was large enough to identify significant correlations.

The closest correlation was observed between MI and DCI, in terms of *P* value and *r*. This suggests potential for shared regulation of circulating concentrations and/or overlap of dietary sources. A possible direct link between MI and DCI concentration within tissues could result from interconversion of MI and DCI, which has been reported to occur via epimerase action (62, 63), although the extent to which this activity occurs in vivo is not well understood. Analysis of plasma DCI in MI-supplemented individuals and vice versa will provide further insight into the relation of circulating MI and DCI.

The relation between glucose and MI status has been unclear. MI can be synthesized from d-glucose via glucose 6-phosphate (Figure 1A), suggesting the potential for positive coregulation of MI and glucose. On the other hand, hyperglycemia is associated with depletion of tissue MI in cultured rodent embryos (64) and diabetes models (65). This could potentially result from competition for the same transporter, SLC5A11 being an example of both an inositol and glucose transporter. We therefore explored the relation between plasma MI and glucose concentration and found a significant positive correlation of MI and glucose concentration in plasma. However, the Spearman *r* value of <0.2 suggests that the plasma MI concentration is not highly dependent on glucose concentration.

Among the other metabolites for which we observed significant correlations in plasma or serum concentrations, the majority of those with higher *r* values are related to the methionine cycle, including methionine itself. These include betaine, which is a methyl donor in the generation of methionine from homocysteine, and its products dimethylglycine and sarcosine (methylglycine) as well as cystathionine, which is generated from homocysteine in the transulfuration pathway. Future studies should examine potential links between inositol and 1-carbon metabolism.

## Supplementary Material

nxac204_Supplemental_Files

## Data Availability

Raw data is available on request from the authors.

## Notes

This study was funded by Sparks and Great Ormond Street Hospital Children's Charity (17ICH03), Action Medical Research (GN2656), and MRC (N003713). The funders had no involvement in study design, data collection and interpretation, writing of the report, or submission of the report for publication.

Author disclosures: The authors report no conflicts of interest.

Supplemental Figures 1–3 and Supplemental Tables 1 and 2 are available from the “Supplementary data” link in the online posting of the article and from the same link in the online table of contents at https://academic.oup.com/jn/.

Abbreviations used: DCI, d*-chiro*-inositol; EGFR, epidermal growth factor receptor; GPI, glycosylphosphatidylinositol; GWAS, genome-wide association study; IMPA1, inositol monophosphatase 1; IP_3_, inositol triphosphate; ISYNA1, inositol-3-phosphate synthase 1; *Itpk1*, inositol 1,3,4-triphosphate 5/6 kinase; LAPTM4B, lysosomal protein transmembrane 4 β; MI, *myo-*inositol; MIOX, *myo*-inositol oxygenase; MTDH, metadherin; NTD, neural tube defect; PIP_2_, phosphatidylinositol (4,5)-biphosphate; SLC5A11, solute carrier family 5 member 11; SMIT2, sodium-dependent *myo*-inositol transporter 2; SNP, single nucleotide polymorphism; TSS, Trinity Student Study; ZP2, zona pellucida 2.
