# Supplementary material for: Investigating Genetic Determinants of Plasma Inositol Status in Adult Humans
Source: J Nutr. 2022 Sep 2;152(11):2333–42. doi: 10.1093/jn/nxac204 (PMC9644178; doi:10.1093/jn/nxac204)
Supplement: nxac204_Supplemental_Files [file nxac204_supplemental_files.zip › Supplementary_Figures_1-3_and_Table_1-Weston_et_al.pdf]

Investigating genetic determinants of plasma inositol status in adult humans. Weston et al.

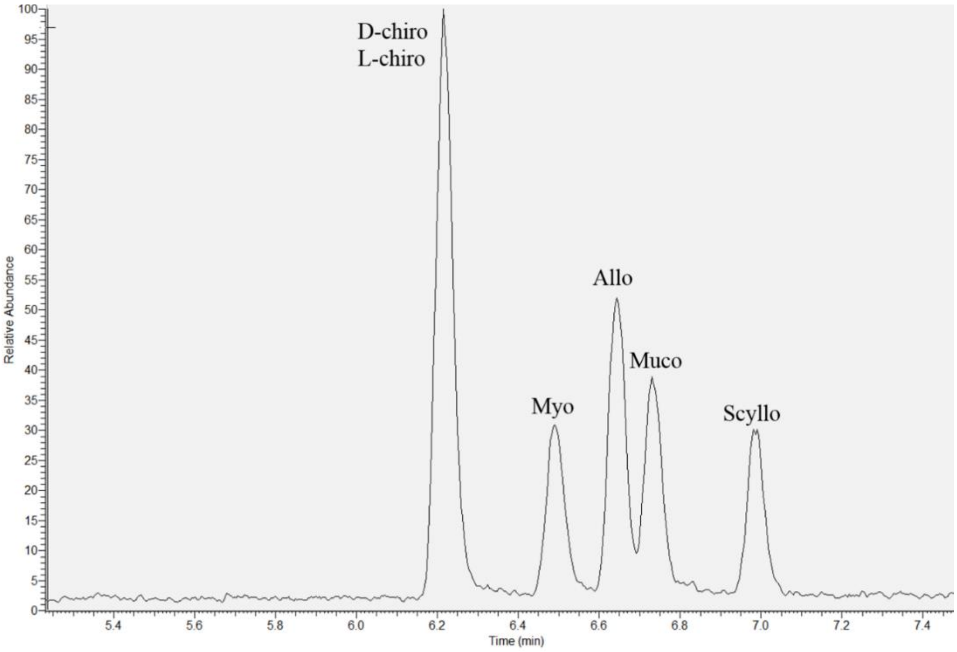

Supplementary Figure 1. Chromatographic separation of inositol isomers.

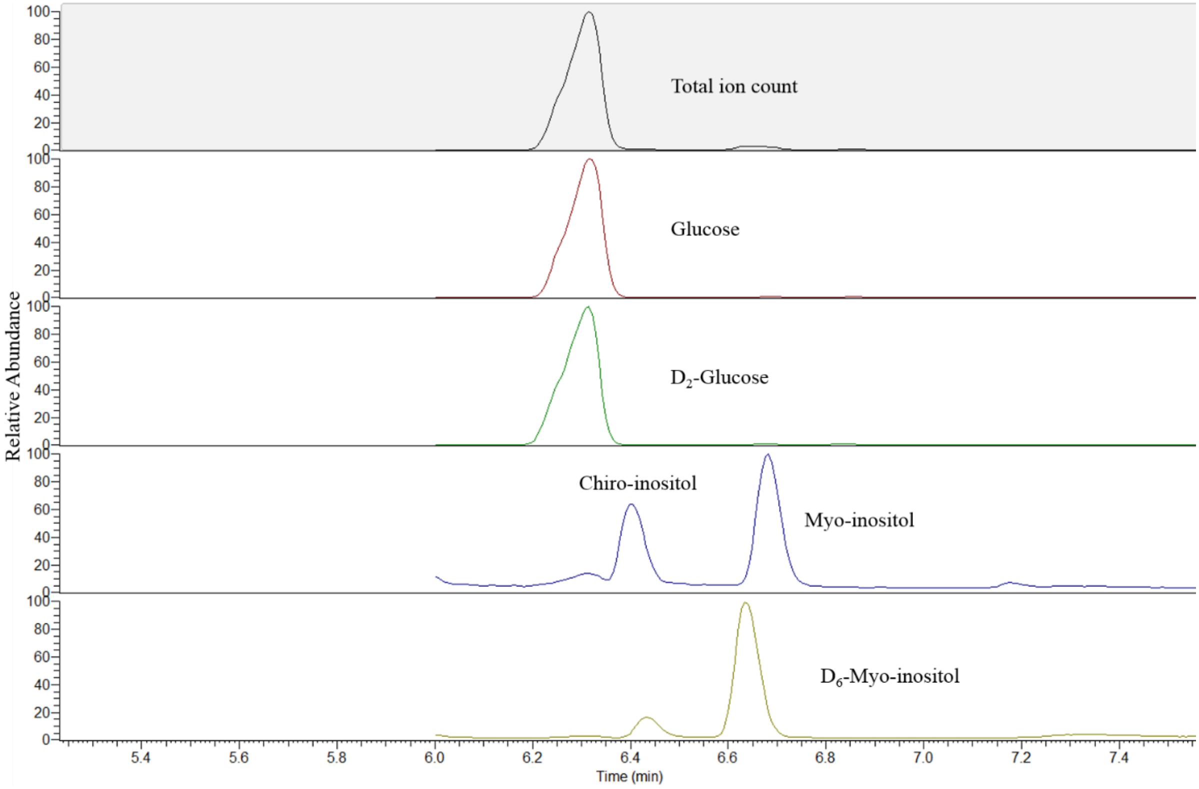

Supplementary Figure 2. Typical chromatogram for glucose and *myo*-inositol. MI and DCI can be distinguished on the basis of differential GC retention time. Glucose is distinguished by different retention time, as well as mass on SRM.

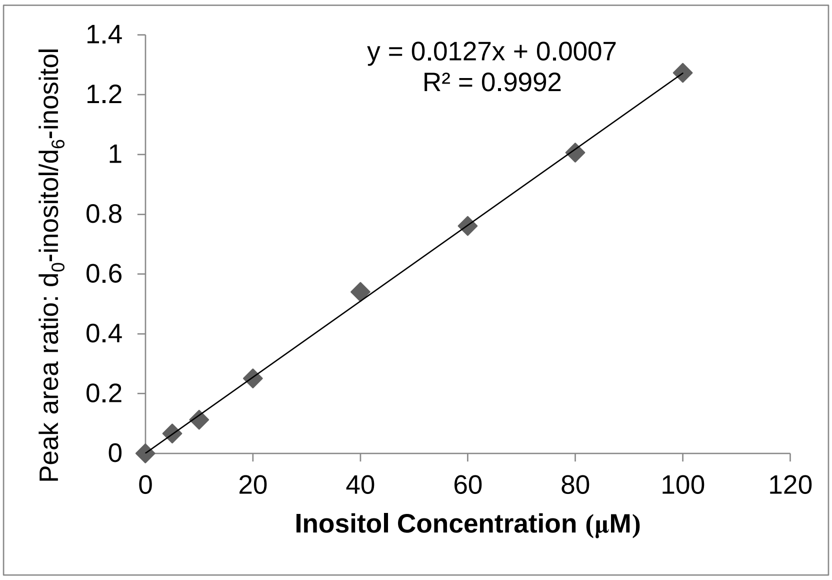

Supplementary Figure 3. Typical standard curve for *myo*-inositol.

Sex	Parameter	*Myo*-inositol (Spearman's ρ)	*P*
**Males & Females**	Height (m)	0.135	1.0×10^{-9} *
	Weight (kg)	0.097	9.2×10^{-6}*
	BMI (kg/m^2)	0.012	0.600
**Males**	Height (m)	0.068	0.016
	Weight (kg)	-0.002	0.936
	BMI (kg/m^2)	-0.048	0.088
**Females**	Height (m)	0.114	0.011
	Weight (kg)	0.062	0.062
	BMI (kg/m^2)	0.055	0.123

Supplementary Table 1. Correlation of plasma *myo*-inositol with age, height and weight. Correlation was determined using raw MI data in the cohort of 2064 individuals (1258 females and 806 males). * Indicates significant correlation ($P<0.0023$ set as level of statistical significance accounting for Bonferroni correction).
