# Supplementary material for: Investigating Genetic Determinants of Plasma Inositol Status in Adult Humans
Source: J Nutr. 2022 Sep 2;152(11):2333–42. doi: 10.1093/jn/nxac204 (PMC9644178; doi:10.1093/jn/nxac204)
Supplement: nxac204_Supplemental_Files [file nxac204_supplemental_files.zip › Supplementary_Table_2-SNPs_analysed_in_candidate_genes_(1).pdf]

Supplementary Table 2

Investigating genetic determinants of plasma inositol status in adult humans.

Weston et al

CHR	SNP	BP	A1	NMISS	BETA	STAT	P	ANNOT
8	RS10102367	82728355	T	2064	0.001024	0.2282	0.8195	.
8	RS11994481	82732439	G	2060	0.0007829	0.1743	0.8616	.
8	RS1058401	82733186	A	2064	0.001383	0.4278	0.6688	IMPA1(0)
8	RS2955014	82733989	T	2063	0.00223	0.5338	0.5935	IMPA1(0)
8	RS2300494	82741285	C	2064	-0.006591	-0.9618	0.3363	IMPA1(0)
8	RS7016327	82744612	G	2064	-0.0001634	-0.0365	0.9709	IMPA1(0)
8	RS1967328	82746326	A	2055	0.001144	0.353	0.7241	IMPA1(0)
8	RS2268431	82749923	A	2057	0.001613	0.3851	0.7002	IMPA1(0)
8	RS204782	82750696	A	2064	0.009819	1.109	0.2674	IMPA1(0)
8	RS204781	82751047	G	2062	0.00568	0.6612	0.5086	IMPA1(0)
8	RS2300490	82752249	G	2047	-0.005811	-0.8443	0.3986	IMPA1(0)
8	RS2912821	82761463	C	2057	-0.0003891	-0.1258	0.8999	.
8	RS2912805	82763795	C	2061	0.001689	0.404	0.6862	.
8	RS2955008	82764401	T	2063	-0.001586	-0.5258	0.5991	.
8	RS10958037	82764511	G	2064	-0.006591	-0.9618	0.3363	.
8	RS1948260	82766797	C	2061	0.001551	0.5292	0.5967	.
8	RS12679058	82767803	C	2059	-0.007219	-1.051	0.2935	.
10	RS1199105	59616678	G	2063	0.002837	0.7318	0.4644	.
10	RS17710296	59625184	T	2064	0.003395	0.4689	0.6392	.
10	RS1698473	59630868	G	2060	0.001096	0.2532	0.8002	IPMK(0)
10	RS11006080	59640631	T	2063	0.01171	0.9877	0.3234	IPMK(0)
10	RS2153281	59642459	A	2062	0.002403	0.6971	0.4858	IPMK(0)
10	RS11006083	59645174	A	2063	-0.00226	-0.5337	0.5936	IPMK(0)
10	RS2590306	59646972	G	2063	0.002502	0.7256	0.4682	IPMK(0)
10	RS2790232	59655677	A	2064	-6.18E-05	-0.006145	0.9951	IPMK(0)
10	RS6481383	59672351	T	2059	0.0007137	0.2369	0.8128	IPMK(0)
10	RS2590350	59684874	C	2054	0.0007548	0.07075	0.9436	IPMK(0)
10	RS2590362	59694118	G	2032	-0.001922	-0.1977	0.8433	IPMK(0)
10	RS7899961	59696431	T	2064	0.009057	1.223	0.2214	IPMK(0)
10	RS1832556	59698254	A	2064	0.002459	0.7135	0.4756	.
10	RS1832557	59698340	A	2064	0.001295	0.2993	0.7648	.
10	RS2251039	59698900	T	2062	0.002445	0.7088	0.4785	CISD1(0)
10	RS1416763	59699169	T	2064	0.002459	0.7135	0.4756	CISD1(0)
10	RS2275442	59705158	T	2062	0.007309	0.939	0.3478	CISD1(0)
12	RS4255600	38432665	G	2064	-0.008904	-2.033	0.04217	.
12	RS7301886	38433866	C	2064	-0.007613	-1.577	0.115	.
12	RS11173433	38434732	T	2064	-0.007613	-1.577	0.115	.
12	RS11173440	38435649	T	2062	-0.00711	-1.467	0.1426	SLC2A13(0)
12	RS11173446	38436953	A	2064	-0.007408	-1.529	0.1263	SLC2A13(0)
12	RS4322461	38437190	T	2063	-0.008862	-2.023	0.04319	SLC2A13(0)
12	RS10735885	38437813	A	2063	-0.008894	-2.03	0.04246	SLC2A13(0)
12	RS10784051	38438158	C	2018	-0.007482	-1.698	0.08961	SLC2A13(0)

12	RS4583047	38438705	C	2063	-0.01381	-1.431	0.1525	SLC2A13(0)
12	RS11173459	38439807	C	2064	-0.007613	-1.577	0.115	SLC2A13(0)
12	RS11173494	38445854	A	2063	-0.007307	-1.512	0.1306	SLC2A13(0)
12	RS11611119	38452524	C	2064	-4.02E-05	-0.01292	0.9897	SLC2A13(0)
12	RS10444570	38455093	C	2060	-0.008717	-1.992	0.04655	SLC2A13(0)
12	RS10747878	38459221	T	2058	-0.008573	-1.959	0.05023	SLC2A13(0)
12	RS11173546	38461138	G	2064	-0.0005392	-0.06821	0.9456	SLC2A13(0)
12	RS4427625	38468873	T	2063	-0.01391	-1.435	0.1514	SLC2A13(0)
12	RS4238078	38479087	T	2035	-0.01488	-1.552	0.1208	SLC2A13(0)
12	RS7966456	38481689	A	2064	-0.005528	-1.808	0.0707	SLC2A13(0)
12	RS4254134	38483076	G	2064	-0.01002	-2.454	0.0142	SLC2A13(0)
12	RS6581351	38484351	A	2063	-0.005524	-1.735	0.08293	SLC2A13(0)
12	RS11173671	38489062	T	2064	0.001026	0.2442	0.8071	SLC2A13(0)
12	RS7294916	38494630	G	2053	-0.0017	-0.5426	0.5875	SLC2A13(0)
12	RS4638371	38498758	A	2062	-0.002012	-0.6444	0.5194	SLC2A13(0)
12	RS10784118	38505614	A	2060	-0.00323	-1.051	0.2935	SLC2A13(0)
12	RS7962260	38510570	A	2064	-0.002306	-0.7357	0.462	SLC2A13(0)
12	RS7963790	38515289	G	2060	0.000999	0.3239	0.746	SLC2A13(0)
12	RS4388962	38521912	G	2054	-0.002194	-0.7499	0.4534	SLC2A13(0)
12	RS4293188	38522359	A	2060	-0.001617	-0.5388	0.5901	SLC2A13(0)
12	RS11173862	38522641	T	2064	-0.003074	-0.5677	0.5703	SLC2A13(0)
12	RS12825170	38527968	T	2060	-0.003037	-0.9128	0.3614	SLC2A13(0)
12	RS7968509	38541115	T	2063	-0.002114	-0.6739	0.5004	SLC2A13(0)
12	RS4429129	38544466	T	2062	-0.002804	-0.9128	0.3615	SLC2A13(0)
12	RS6581405	38550814	A	2062	-0.0003965	-0.13	0.8965	SLC2A13(0)
12	RS7967517	38551053	C	2063	0.002988	0.6828	0.4948	SLC2A13(0)
12	RS4423249	38554997	C	2064	-0.003917	-1.326	0.185	SLC2A13(0)
12	RS4277185	38559489	T	2062	0.00152	0.4784	0.6324	SLC2A13(0)
12	RS12310523	38563353	A	2063	0.002679	0.7335	0.4633	SLC2A13(0)
12	RS12312400	38567960	A	2062	0.001892	0.5985	0.5496	SLC2A13(0)
12	RS884864	38570933	C	2064	0.00327	0.9138	0.3609	SLC2A13(0)
12	RS11174153	38571703	G	2062	-0.001847	-0.6258	0.5315	SLC2A13(0)
12	RS953493	38572369	G	2063	-0.01347	-1.931	0.05363	SLC2A13(0)
12	RS10784261	38574200	G	2041	0.003662	1.25	0.2113	SLC2A13(0)
12	RS956066	38583007	A	2063	0.00332	1.135	0.2563	SLC2A13(0)
12	RS12817755	38585079	T	2064	-7.86E-05	-0.02256	0.982	SLC2A13(0)
12	RS11612814	38588866	A	2063	-0.00254	-0.8357	0.4034	SLC2A13(0)
12	RS11833368	38589772	C	2062	0.005583	1.915	0.05568	SLC2A13(0)
12	RS17127259	38589898	A	2064	0.002152	0.3833	0.7015	SLC2A13(0)
12	RS10877772	38594240	A	2064	0.00564	1.932	0.05344	SLC2A13(0)
12	RS2088354	38596875	T	2060	-0.01055	-1.266	0.2056	SLC2A13(0)
12	RS11174281	38597760	T	2063	0.003043	0.888	0.3747	SLC2A13(0)
12	RS10506138	38598763	T	2064	0.000585	0.1302	0.8964	SLC2A13(0)
12	RS6581439	38608113	T	2063	-0.00273	-0.6492	0.5163	SLC2A13(0)
12	RS17127277	38609091	G	2064	0.006559	0.9004	0.368	SLC2A13(0)
12	RS11174343	38610573	C	2063	0.003323	0.8282	0.4077	SLC2A13(0)

12	RS4767956	38610941	G	2063	-0.000157	-0.05253	0.9581	SLC2A13(0)
12	RS10506139	38614403	C	2061	0.004565	0.559	0.5762	SLC2A13(0)
12	RS7969011	38614951	A	2062	0.008455	1.137	0.2557	SLC2A13(0)
12	RS7972838	38615024	T	2064	0.008466	1.139	0.2548	SLC2A13(0)
12	RS11174427	38629971	T	2063	-0.002866	-0.5703	0.5685	SLC2A13(0)
12	RS28370798	38630473	T	2064	0.004532	0.5552	0.5788	SLC2A13(0)
12	RS11564225	38631920	C	2064	0.004532	0.5552	0.5788	SLC2A13(0)
12	RS10877832	38632988	G	2064	0.0009368	0.2003	0.8413	SLC2A13(0)
12	RS12296462	38635503	A	2063	0.003798	0.5317	0.595	SLC2A13(0)
12	RS1390952	38635770	A	2064	0.002982	0.9323	0.3513	SLC2A13(0)
12	RS10506140	38636641	G	2063	-0.00157	-0.5328	0.5942	SLC2A13(0)
12	RS12582658	38637109	G	2064	0.008466	1.157	0.2476	SLC2A13(0)
12	RS2896824	38637192	A	2064	0.007771	1.743	0.0815	SLC2A13(0)
12	RS7312341	38637298	G	2064	-0.001578	-0.4756	0.6344	SLC2A13(0)
12	RS10877835	38637759	A	2064	0.003639	0.9057	0.3652	SLC2A13(0)
12	RS7973509	38640414	A	2063	-0.001377	-0.4142	0.6788	SLC2A13(0)
12	RS11174478	38640511	A	2061	-0.001455	-0.4919	0.6228	SLC2A13(0)
12	RS549790	38642537	G	2056	-0.008399	-1.163	0.245	SLC2A13(0)
12	RS545385	38642980	A	2064	-0.0101	-1.463	0.1435	SLC2A13(0)
12	RS574918	38643914	A	2040	-0.002181	-0.5646	0.5724	SLC2A13(0)
12	RS10877867	38648543	A	2061	-0.003095	-0.8873	0.375	SLC2A13(0)
12	RS7976837	38651603	A	2064	-0.001903	-0.6231	0.5333	SLC2A13(0)
12	RS481241	38653395	T	2062	0.00745	1.863	0.06267	SLC2A13(0)
12	RS12810368	38653438	C	2063	-0.003318	-0.9642	0.335	SLC2A13(0)
12	RS7311756	38656440	A	2064	0.003384	0.7469	0.4552	SLC2A13(0)
12	RS992851	38657759	A	2064	-0.003848	-0.7873	0.4312	SLC2A13(0)
12	RS7138679	38664914	T	2064	-0.003854	-1.269	0.2046	SLC2A13(0)
12	RS11564282	38665006	A	2062	-0.004055	-0.8288	0.4073	SLC2A13(0)
12	RS12824918	38665040	T	2063	-0.004182	-1.242	0.2142	SLC2A13(0)
12	RS12423473	38666068	A	2060	-0.004721	-1.38	0.1677	SLC2A13(0)
12	RS556227	38666789	T	2063	0.005301	1.192	0.2333	SLC2A13(0)
12	RS11174609	38667030	T	2064	-0.001234	-0.4189	0.6753	SLC2A13(0)
12	RS1086490	38668387	C	2062	-0.00434	-0.8906	0.3733	SLC2A13(0)
12	RS555740	38668966	C	2064	-0.003703	-1.224	0.221	SLC2A13(0)
12	RS11174631	38671385	T	2064	0.002662	0.2705	0.7868	SLC2A13(0)
12	RS7964150	38674604	G	2063	0.002488	0.8387	0.4018	SLC2A13(0)
12	RS28370643	38680119	T	2064	-0.008361	-0.7464	0.4555	SLC2A13(0)
12	RS28370644	38683502	C	2060	-0.0003682	-0.08837	0.9296	SLC2A13(0)
12	RS515205	38689229	G	2063	-0.004485	-1.388	0.1652	SLC2A13(0)
12	RS515291	38689259	G	2063	-0.002899	-0.947	0.3438	SLC2A13(0)
12	RS11174735	38692779	C	2064	0.001567	0.346	0.7294	SLC2A13(0)
12	RS2242367	38699965	A	2060	-0.0006814	-0.2093	0.8343	SLC2A13(0)
12	RS1994090	38714828	G	2056	-0.004609	-1.276	0.2022	SLC2A13(0)
12	RS2404347	38719542	G	2064	-0.002482	-0.4825	0.6295	SLC2A13(0)
12	RS11174928	38726750	A	2061	0.002468	0.4245	0.6712	SLC2A13(0)
12	RS10784356	38729243	T	2062	0.002414	0.8096	0.4183	SLC2A13(0)

12	RS10784359	38732017	T	2064	0.002637	0.8907	0.3732	SLC2A13(0)
12	RS7134524	38739548	A	2064	-0.003799	-0.9337	0.3506	SLC2A13(0)
12	RS7971218	38746794	G	2059	0.0005853	0.1466	0.8834	SLC2A13(0)
12	RS7974495	38747205	G	2063	-0.001767	-0.3437	0.7311	SLC2A13(0)
12	RS11175027	38750776	T	2062	0.001519	0.3355	0.7373	SLC2A13(0)
12	RS12823555	38750970	T	2062	0.001981	0.6633	0.5072	SLC2A13(0)
12	RS4768212	38760414	C	2063	-0.001397	-0.2298	0.8183	SLC2A13(0)
12	RS11175094	38761145	G	2060	0.001206	0.2656	0.7906	SLC2A13(0)
12	RS2708453	38764919	T	2064	-0.003679	-0.8964	0.3701	SLC2A13(0)
12	RS2896905	38779683	A	2063	0.002298	0.7686	0.4422	SLC2A13(0)
12	RS17483628	38784457	A	2064	-0.0006031	-0.1501	0.8807	SLC2A13(0)
12	RS17442511	38784950	G	2063	-0.003488	-0.678	0.4979	SLC2A13(0)
12	RS949640	38789953	C	2063	0.002497	0.4476	0.6545	.
14	RS2295396	92464840	G	2064	0.001627	0.4503	0.6526	CHGA(0)
14	RS9658655	92467784	C	2058	-0.001431	-0.2837	0.7766	CHGA(0)
14	RS9658656	92467831	A	2063	-0.001779	-0.3529	0.7242	CHGA(0)
14	RS9658664	92468602	A	2057	-0.008265	-0.5759	0.5648	CHGA(0)
14	RS729940	92468854	T	2040	-0.00096	-0.2359	0.8135	CHGA(0)
14	RS750678	92470632	C	2063	-0.004331	-1.424	0.1546	CHGA(0)
14	RS941581	92470931	T	2064	0.001944	0.5919	0.554	CHGA(0)
14	RS2281514	92473513	G	2064	0.002472	0.854	0.3932	.
14	RS6575304	92474427	T	2064	0.002653	0.9169	0.3593	.
14	RS8011546	92474863	G	2048	0.002653	0.9136	0.361	.
14	RS1043542	92476815	T	2063	0.002278	0.7558	0.4499	ITPK1(0)
14	RS11446	92477001	T	2063	0.005352	1.747	0.08075	ITPK1(0)
14	RS4905011	92478204	A	2062	-0.001291	-0.3772	0.706	ITPK1(0)
14	RS3783925	92481657	C	2063	0.0003916	0.1286	0.8977	ITPK1(0)
14	RS2295394	92482496	T	2061	0.0004009	0.0763	0.9392	ITPK1(0)
14	RS4900164	92486689	G	2063	0.004365	1.286	0.1985	ITPK1(0)
14	RS1006888	92488705	C	2064	-0.0001668	-0.0429	0.9658	ITPK1(0)
14	RS2402226	92489288	C	2064	0.001892	0.6342	0.526	ITPK1(0)
14	RS4586354	92495041	C	2063	0.0097	1.69	0.09111	ITPK1(0)
14	RS11625662	92495492	A	2062	-0.003959	-0.9291	0.353	ITPK1(0)
14	RS941578	92496373	G	2064	-0.0007904	-0.1512	0.8798	ITPK1(0)
14	RS4905017	92508574	C	2064	0.005349	1.363	0.173	ITPK1(0)
14	RS7154944	92520221	T	2060	0.003531	0.8791	0.3795	ITPK1(0)
14	RS12589455	92526306	G	2064	0.001003	0.2683	0.7885	ITPK1(0)
14	RS3783913	92527504	G	2061	-0.0003222	-0.1065	0.9152	ITPK1(0)
14	RS12435423	92529612	C	2064	0.002605	0.5676	0.5704	ITPK1(0)
14	RS12586382	92533771	A	2063	0.001538	0.3416	0.7327	ITPK1(0)
14	RS4905029	92538684	T	2064	-0.001996	-0.5686	0.5697	ITPK1(0)
14	RS17128737	92541968	A	2062	-0.006266	-1.161	0.2456	ITPK1(0)
14	RS3783910	92546482	C	2064	-0.0006476	-0.187	0.8517	ITPK1(0)
14	RS3783903	92552076	G	2064	-0.002877	-0.8199	0.4124	ITPK1(0)
14	RS11628021	92555119	T	2064	-0.003073	-0.6819	0.4954	ITPK1(0)
14	RS10144329	92557405	C	2063	-0.003498	-1.139	0.2549	ITPK1(0)

14	RS1740696	92567902	G	2063	-0.00406	-1.326	0.1851	ITPK1(0)
14	RS1740598	92568834	A	2064	8.45E-05	0.01901	0.9848	ITPK1(0)
14	RS1740596	92576559	T	2063	0.0007849	0.2654	0.7908	ITPK1(0)
14	RS2180369	92586218	G	2063	0.002603	0.5836	0.5595	ITPK1(0)
14	RS941542	92593330	G	2064	-0.001075	-0.3631	0.7166	ITPK1(0)
14	RS749619	92593647	A	2064	0.004248	0.9665	0.3339	ITPK1(0)
14	RS2749509	92597867	G	2064	-0.002923	-0.8776	0.3803	ITPK1(0)
14	RS882023	92601767	C	2059	3.59E-05	0.01192	0.9905	ITPK1(0)
14	RS2236131	92610659	A	2063	-0.001338	-0.4159	0.6775	ITPK1(0)
14	RS1740698	92618491	T	2064	0.001152	0.3603	0.7187	ITPK1(0)
14	RS8019022	92619230	C	2064	0.006316	1.526	0.1272	ITPK1(0)
14	RS4905043	92619762	A	2060	0.001476	0.492	0.6228	ITPK1(0)
14	RS11845823	92623469	G	2064	0.006882	2.002	0.04547	ITPK1(0)
14	RS941536	92631173	C	2064	0.009567	0.8133	0.4162	ITPK1(0)
14	RS957362	92635569	C	2064	0.007731	2.218	0.02663	ITPK1(0)
16	RS2343605	24756164	G	2063	0.006808	2.248	0.02468	.
16	RS7195553	24760616	A	2064	-0.001519	-0.4776	0.633	.
16	RS2667747	24767551	G	2059	-0.003778	-1.128	0.2594	SLC5A11(0)
16	RS8051275	24769228	T	2063	0.005421	1.023	0.3065	SLC5A11(0)
16	RS8054332	24769428	T	2064	-0.0002659	-0.05596	0.9554	SLC5A11(0)
16	RS274115	24771208	G	2058	0.005106	1.416	0.157	SLC5A11(0)
16	RS1549238	24772926	C	2064	-0.003498	-1.162	0.2452	SLC5A11(0)
16	RS8062004	24779915	G	2063	-0.005566	-1.429	0.1531	SLC5A11(0)
16	RS274103	24783646	A	2064	9.82E-05	0.03304	0.9736	SLC5A11(0)
16	RS274108	24786457	A	2049	0.001313	0.4527	0.6508	SLC5A11(0)
16	RS2547039	24790674	G	2062	0.004133	1.225	0.2207	SLC5A11(0)
16	RS11862551	24795348	T	2063	0.00391	0.8498	0.3955	SLC5A11(0)
16	RS274073	24795616	A	2063	-0.001939	-0.4035	0.6866	SLC5A11(0)
16	RS8051759	24795643	A	2063	0.002411	0.7946	0.4269	SLC5A11(0)
16	RS8052587	24796094	G	2064	0.003679	0.8095	0.4183	SLC5A11(0)
16	RS1011078	24801178	G	2064	0.004821	1.639	0.1013	SLC5A11(0)
16	RS7186357	24801769	G	2061	0.005241	1.691	0.09105	SLC5A11(0)
16	RS17178242	24806089	A	2063	0.001463	0.4957	0.6201	SLC5A11(0)
16	RS274068	24806473	C	2064	-0.0002267	-0.06279	0.9499	SLC5A11(0)
16	RS274077	24808794	C	2064	0.002368	0.6803	0.4964	SLC5A11(0)
16	RS274082	24809935	C	2064	0.002161	0.6202	0.5352	SLC5A11(0)
16	RS274098	24819373	C	2063	0.002294	0.6961	0.4865	SLC5A11(0)
16	RS13338696	24823496	A	2064	-0.002049	-0.443	0.6578	SLC5A11(0)
16	RS12443852	24824845	G	2064	0.003284	1.064	0.2875	SLC5A11(0)
16	RS13339160	24825198	T	2064	0.003284	1.064	0.2875	SLC5A11(0)
16	RS17772929	24825549	T	2064	-0.002123	-0.7269	0.4674	SLC5A11(0)
16	RS17178990	24829446	A	2064	-0.01901	-2.95	0.00321	SLC5A11(0)
16	RS4788437	24831925	T	2063	0.003181	1.068	0.2858	.
16	RS11647538	24836976	C	2064	0.003437	0.7818	0.4344	.
16	RS28540434	24838049	C	2063	-0.02035	-3.356	0.0008065	.
16	RS4788439	24839299	T	2063	-0.01953	-3.184	0.001474	ARHGAP17(0)

16	RS13143	31396534	T	2062	-0.0009213	-0.2814	0.7784	TGFB1I1(0)
16	RS11150626	31399483	C	2061	-0.0007923	-0.2423	0.8086	.
16	RS3116150	31405522	A	2063	0.007742	2.268	0.02343	SLC5A2(0)
16	RS9927250	31414401	A	2063	-0.001915	-0.5174	0.605	C16orf58(0)
16	RS11865835	31417317	C	2063	-0.0003834	-0.1173	0.9066	C16orf58(0)
19	RS10405636	18399742	C	2040	0.00639	2.163	0.03065	SSBP4(0)
19	RS888669	18401644	T	2064	0.007427	2.153	0.03141	SSBP4(0)
19	RS10409003	18404054	T	2057	0.006453	2.159	0.03098	SSBP4(0)
19	RS10442	18406341	C	2064	0.006827	2.301	0.02147	SSBP4(0)
19	RS2303697	18407678	G	2060	0.006973	2.346	0.01908	ISYNA1(0)
19	RS10427083	18411276	A	2063	0.007192	2.425	0.01539	.
19	RS12981988	18413139	A	2064	0.005797	0.9587	0.3378	.
21	RS4817616	34366368	A	2064	0.002278	0.7631	0.4455	.
21	RS10854369	34380835	G	2064	-0.004518	-0.7383	0.4604	SLC5A3(0)|MRPS6(0)
21	RS2834375	34381492	G	2064	0.000461	0.1518	0.8793	SLC5A3(0)|MRPS6(0)
21	RS2834377	34384533	C	2060	-0.004114	-0.6719	0.5017	SLC5A3(0)|MRPS6(0)
21	RS1782995	34388613	C	2063	-0.005259	-0.846	0.3977	SLC5A3(0)|MRPS6(0)
21	RS2211792	34393685	T	2062	0.0001337	0.04386	0.965	SLC5A3(0)|MRPS6(0)
21	RS2244724	34396280	G	2064	0.0005859	0.1928	0.8471	SLC5A3(0)|MRPS6(0)
21	RS2032107	34396474	C	2064	-4.16E-05	-0.006526	0.9948	SLC5A3(0)|MRPS6(0)
21	RS9984245	34399933	T	2062	-0.004101	-1.06	0.2892	SLC5A3(0)|MRPS6(0)
22	RS130261	30763824	G	2025	0.003118	1.043	0.297	.
22	RS130262	30765409	G	2057	0.003885	1.323	0.1858	.
22	RS738993	30767340	A	2063	-0.001756	-0.3718	0.7101	.
22	RS135023	30772776	A	2064	0.00377	1.283	0.1995	SLC5A1(0)
22	RS130264	30774217	A	2064	-0.001556	-0.4213	0.6735	SLC5A1(0)
22	RS17683011	30775946	G	2063	0.007985	1.474	0.1405	SLC5A1(0)
22	RS17745316	30776805	A	2063	0.002678	0.5978	0.5501	SLC5A1(0)
22	RS9606899	30777811	C	2064	0.008719	2.023	0.04316	SLC5A1(0)
22	RS130266	30780362	C	2064	0.003568	1.218	0.2234	SLC5A1(0)
22	RS9609420	30789994	G	2064	0.0003673	0.08369	0.9333	SLC5A1(0)
22	RS9609421	30791497	G	2064	0.009416	1.263	0.2069	SLC5A1(0)
22	RS739009	30797651	C	2064	-0.002135	-0.7295	0.4658	SLC5A1(0)
22	RS7287435	30806600	A	2064	0.007311	0.639	0.5229	SLC5A1(0)
22	RS17683430	30817700	A	2059	0.007841	1.446	0.1482	SLC5A1(0)
22	RS17683448	30817744	T	2064	0.007936	1.465	0.1429	SLC5A1(0)
22	RS17683704	30836041	G	2063	0.007934	1.465	0.1432	SLC5A1(0)
22	RS9609427	30839440	A	2064	-0.001572	-0.4271	0.6693	.
22	RS9606905	30846261	T	2054	-0.0007431	-0.2047	0.8378	.
22	RS9616854	49265272	A	2063	-0.001095	-0.3744	0.7081	.
22	RS13056677	49268510	A	2061	0.005818	0.9523	0.341	ADM2(0)
22	RS2236031	49268560	T	2031	0.002122	0.5599	0.5756	ADM2(0)
22	RS13055726	49269410	C	2064	0.001717	0.3718	0.7101	ADM2(0)
22	RS876278	49269908	C	2042	-0.005881	-1.016	0.3099	ADM2(0)
22	RS761745	49270083	T	2058	0.004264	1.378	0.1685	ADM2(0)
22	RS2232873	49271964	A	2063	-0.001855	-0.3024	0.7624	.

22	RS2232879	49272637	A	2064	-4.61E-05	-0.009604	0.9923	MIOX(0)
22	RS1055271	49275206	C	2057	0.003171	1.055	0.2917	MIOX(0)
22	RS4824157	49276447	C	2059	0.0001471	0.04145	0.9669	.
22	RS134974	49282402	G	2063	-0.005465	-1.871	0.0615	.
22	RS8138406	49284311	C	2063	-0.00466	-1.593	0.1114	.
22	RS28375115	49285480	C	2064	0.0006367	0.1015	0.9191	.

Supplementary Table 2. List of SNPs analysed in candidate gene regions. CHR, chromosome; SNP, SNP ID; BP, physical position (base-pair); A1, minor allele; NMISS, number of non-missing individuals; BETA, regression coefficient; STAT, standard chi-squared statistics; P, asymptotic p-value; ANNOT, gene annotation**.** P-values below 0.05 are highlighted in yellow.
